# Frequency of Bioprosthetic Valve Fracturing Utilization in an All-Comers Valve-in-Valve TAVR Cohort

**DOI:** 10.3389/fcvm.2021.653871

**Published:** 2021-07-01

**Authors:** Hendrik Ruge, Magdalena Erlebach, Ruediger Lange

**Affiliations:** ^1^Department of Cardiovascular Surgery, German Heart Centre Munich, Technical University Munich, Munich, Germany; ^2^German Heart Center Munich, Institute for Translational Cardiac Surgery, Technical University Munich, Munich, Germany; ^3^Partner Site Munich Heart Alliance, German Centre for Cardiovascular Research (DZHK), Munich, Germany

**Keywords:** bioprostethic valve fracturing, transprosthetic gradient, postdilatation, valve in valve transcatheter aortic valve implantation, surgical aortic valve

## Abstract

**Introduction:** Valve-in-valve TAVR (ViV-TAVR) is an established treatment for failing surgical aortic valves in patient at high surgical risk. Elevated transprosthetic gradients are common after ViV-TAVR. Previously, bench tests showed feasibility of bioprosthetic valve fracturing (VF) using high-pressure balloons. Small case series show reduced transprosthetic gradients using VF. We present our clinical experience and outcome of VF.

**Material and Methods:** Consecutive ViV-TAVR patients were identified from our institutional TAVR database and utilization of bioprosthetic valve fracturing or intraprocedural postdilatation was reviewed. Surgical valves were categorized as responsive or not responsive to VF. Transprosthetic gradients were compared in procedures with VF and procedures with or without postdilatation.

**Results:** In 67 consecutive ViV-TAVR procedures between January 2018 and September 2020, VF was attempted in 15 cases with eight being successful. Standard postdilatation was performed in 21 patients and 31 cases were without postdilatation. Mean transprosthetic gradients (MPG) decreased from 34.2 + 12.5 to 12.7 + 7.4 mmHg (*p* < 0.001) for all patients. MPG was 8.6 + 3.5 mmHg after VF, 12.9 + 8.5 mmHg after standard postdilatation (*p* = 0.18) and 13.4 + 6.8 mmHg in cases without postdilatation (*p* = 0.04). In small surgical valves with true inner diameter <21 mm MPG was 9.1 + 3.5 mmHg after VF, 14.2 + 8.9 after standard postdilatation (*p* = 0.068) and 16.2 + 9.2 mmHg without postdilatation (*p* = 0.152). Failed attempts with BVF occurred with the Perimount standard valve.

**Conclusion:** Bioprosthetic valve fracturing results in lower mean transprosthetic gradients after ViV-TAVR. Responsiveness of BVF in Perimount surgical valves, long-term hemodynamic outcome, and potential survival benefits need further evaluation.

## Introduction

Valve-in-valve transcatheter aortic valve replacement (ViV-TAVR) has evolved as an alternative to reoperation for patients presenting with failing surgical aortic valves (1, 2). In small surgical aortic valves with a true inner diameter ≤21 mm, elevated transprosthetic gradients >20 mmHg are found in up to 40% after ViV-TAVR and may contribute to the worse 1-year and long-term survival of these patients compared to patients undergoing ViV-TAVR with larger surgical valves (3, 4).

Bioprosthetic valve fracturing (BVF) of a surgical aortic valve was first described in 2015 (5). High-pressure balloons are used to mechanically fracture the surgical bioprosthetic valve either before or after implantation of a transcatheter heart valve (THV). This technique aims to support optimal frame expansion of the THV or even allows implantation of a larger THV by increasing the effective orifice area of the failing surgical valve. Thereby, lower transprosthetic gradients after ViV-TAVR could potentially be achieved.

Bench tests proved the feasibility of the concept and identified valves that could successfully be fractured (5–8). Surgical valves responsive to BVF are Biocor Epic (Abbott), Mosaic (Medtronic), Magna (Edwards Lifesciences), Magna ease (Edwards Lifesciences), Mitroflow (Sorin), and “newer generation” Perimount (Edwards Lifesciences). Surgical valves that are not responsive to BVF are Hancock II and Avalus (both Medtronic). Valves that are not responsive to BVF but allow “remodeling” are the Trifecta (Abbott), Inspiris, Carpentier-Edwards, and Carpentier-Edwards supra-annular and “older-generation” Perimount valves (all Edwards Lifesciences) (5–9).

Especially in small surgical aortic valves, BVF is considered to improve transvalvular gradients, whereas the effect is unclear in larger surgical valves (10, 11).

The clinical application of the valve fracturing technology, as well as the rate of successful fracturing of surgical valves in ViV-TAVR procedures, is not well-described.

This study aims to report on the clinical application of BVF in consecutive, single-center ViV-TAVR procedures. Furthermore, we analyzed how often valves, for which successful fracturing has been shown *in vitro*, could be fractured *in vivo*.

## Materials and Methods

Consecutive ViV-TAVR procedures from January 2018 to September 2020 were identified from our institutional TAVR database.

Prospectively collected data was retrospectively analyzed. The local ethics committee approved the study (647/20 S).

The true inner diameter (ID) of the surgical valves was obtained from “ViV Aortic” application developed by UBQO Ltd. and Dr. Vinayak Bapat and computerized tomographies.

The primary endpoint was clinical application and technical success of BVF.

Secondary endpoints were postprocedural transprosthetic gradients acquired by echocardiography after ViV-TAVR with and without VF. Prosthetic valve function after ViV-TAVR, intra-hospital survival, vascular complications, bleeding complications, and stroke were reported according to VARC-2 criteria (12).

The decision for standard postdilatation or bioprosthetic ring fracturing was based on the size of the surgical valve, extent of frame expansion of the THV, responsiveness of the surgical valve to valve fracturing, and patient comorbidities.

### BVF

In all procedures, ViV-TAVR was executed prior to BVF. After THV implantation, a postdilatation with a high-pressure balloon (True Dilatation balloon or Atlas Gold balloon, C.R. Bard, Murray Hill, NJ, USA), at least 1 mm larger than the true ID of the surgical valve, was performed under rapid ventricular pacing. A 40-ml inflator was used to fill the balloon with diluted contrast and increase the pressure reaching 15–18 atm. Successful BVF was indicated by a sudden pressure drop in the inflator often accompanied by an audible bump and a full cylindrical balloon shape without a waist at the level of the bioprosthetic ring. The labeled outer diameter of the balloon in relation to the ID of the surgical valve was expressed as a ratio.

### Postdilatation

Postdilatation was mainly performed using a noncompliant balloon (True Dilatation balloon or Atlas Gold balloon, C.R. Bard, Murray Hill, NJ, USA) either due to underexpansion of the THV frame or due to optimization of THV frame expansion within a surgical valve known to resist BVF (i.e., Trifecta, St. Jude Medical, Minneapolis, MN, USA). In two procedures, the balloon provided with the Edwards Sapien THV and in one case a NuCLEUS balloon (NuMED Inc., Hopkinton, USA) were used. For hemodynamic analysis, patients with failed valve fracturing attempts were included in the “postdilatation” group.

### Statistical Analysis

Statistical analysis was conducted using JASP (JASP Team 2020, Version 0.13.1). Continuous variables are presented as mean ± standard deviation or as median (interquartile range); categorical variables are expressed as percentages. Comparison between groups was performed using either a Fisher exact test for binominal variables, a *t*-test for normal distributed variables, or a Wilcoxon rank-sum test for the remaining variables. A *p*-value of < 0.05 was considered as significant.

## Results

At our center, the first BVF was performed in April 2018. From January 2018 to September 2020, 67 consecutive patients underwent ViV-TAVR procedures (Table 1). Of these, BVF was attempted in 15 cases with eight being successful (Table 2). Intraprocedural postdilatation of the bioprosthetic valve was performed in 21 cases, including 16 ViV-TAVR with a Trifecta surgical valve (St. Jude Medical, Minneapolis, MN, USA). Thirty-one ViV-TAVR procedures were performed without postdilatation.

**Table 1:** Baselines characteristic of the ViV-TAVR cohort.

Age, year ± SD	72 + 10
Female gender, *n* (%)	19 (28%)
BMI	28 ± 13.8
Time from SAVR to TAVR, year ± SD	9.1 + 3.1
Log EuroSCORE, %±SD	20.0 + 16.3
EuroSCORE II, ±SD	8.7 + 10.7
STS PROM, %±SD	4.6 + 7.1
Transprosthetic gradient max, mmHg ± SD	58 + 19
Transprosthetic gradient mean, mmHg ± SD	34 + 13
Regurgitation	
None/mild, *n* (%)	37 (55%)
Moderate, *n* (%)	12 (18%)
Severe, *n* (%)	18 (27%)
Internal diameter surgical valve, mm ± SD	21.7 ± 2.5
Coronary artery disease, *n* (%)	32 (48%)
Previous CABG, *n* (%)	15 (22%)
Previous stroke, *n* (%)	6 (9%)
Atrial fibrillation, *n* (%)	13 (19%)
Previous permanent pacemaker, *n* (%)	15 (22%)
Left ventricular ejection fraction	
>50%, *n* (%)	43 (64%)
>35–50%, *n* (%)	19 (28%)
<35%, *n* (%)	5 (7%)
Pulmonary disease, *n* (%)	11 (16%)
Pulmonary hypertension, PAP sys. > 60 mmHg, *n* (%)	6 (9%)

**Table 2:** Procedural details of bioprosthetic ring fracturing attempts.

**Successful ring fracturing**	**Surgical valve**	**Time to ViV-TAVR**	**True ID**	**THV**	**Pre ViV-TAVR mean gradient**	**High-pressure balloon**	**Balloon–ID ratio**	**Post ViV-TAVR mean gradient**
Y	Mosaic 23	134	19	Evolut 26	20	TRU 24	1.26	6
Y	Perimount 23	148	21	Evolut 26	12	TRU 24	1.14	12
Y	Perimount 23	166	21	Evolut 26	39	TRU 24	1.14	10
Y	Magna ease 21	140	19	Evolut 23	25	TRU 22	1.16	9
Y	Mosaic 27	105	22	Evolut 26	22	TRU 24	1.09	5
Y	Perimount 23	245	21	Acurate neo S	28	TRU 22	1.05	12
Y	Magna ease 21	121	19	Evolut 23	44	TRU 22	1.16	3
Y	Perimount 21	148	19	Evolut 23	28	TRU 22	1.16	12
N	Perimount 21	115	19	Evolut 23	34	TRU 20	1.05	7
N	Perimount 25	160	23	Evolut 29	45	TRU 26	1.13	11.5
N	Perimount 25	153	23	Sapien ultra 26	30	TRU 25	1.09	11
N	Perimount 27	123	25	Sapien ultra 26	39	TRU 26	1.04	14
N	Perimount 25	160	23	Evolut 29	Severe insufficiency	TRU 25	1.09	3.5
N	Perimount 21	127	19	Evolut 23	33	TRU 24	1.26	18
N	Perimount 21	59	19	Evolut 23	severe insufficiency	TRU 22	1.16	7

*All Perimount valves were of the P2800 model*.

The mean age was 72 ± 10 years with 19 female patients. The mean STS score was 4.6 ± 7.1, and the mean log EuroSCORE was 20.0 ± 16.3%. The time from surgical aortic valve replacement to ViV-TAVR was 9.1 ± 3.1 years.

### Characteristics of Surgical Valves

The mean true ID of the degenerated surgical valves was 21.7 ± 2.5 mm (range 17–27 mm).

**Figure 1:**
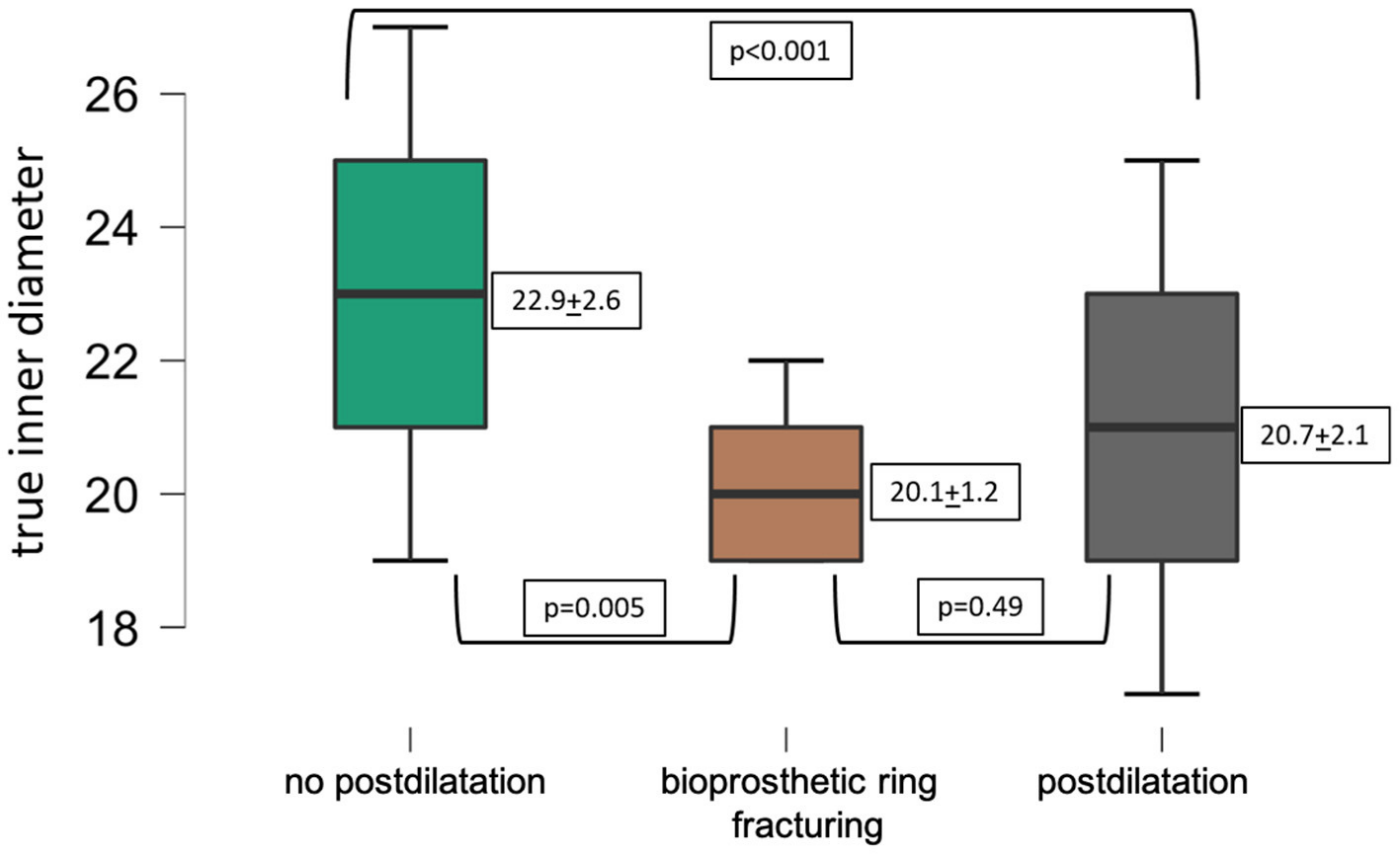
Mean true inner diameter of failing surgical valves.

The ID of surgical valves was smaller in procedures where BVF or postdilatation was performed compared to the ID of surgical valves in procedures without postdilatation (Figure 1).

### Procedural Details

Transcatheter valve-in-valve procedures were performed with Evolut R (Medtronic, Minneapolis, MN, USA) in 53 cases, Sapien 3/Sapien 3 ultra (Edwards Lifesciences, Irvine, CA, USA) in 10 cases, and Acurate neo (Boston Scientific, Marlborough, MA, USA) in four cases. Vascular access was transfemoral in 62 cases, transapical in three, transaxillary in one, and transaortic in one case. Cerebral protection was used in five cases.

Of the failing surgical valves, 39 were potentially responsive to BVF, 25 were known to resist BVF, and for three valves (all BioValsalva conduit, Vascutek, Inchinnan, UK) no information was available on responsiveness to BVF attempts (Table 3).

**Table 3:** Failing surgical aortic valves treated with ViV-TAVR.

**Surgical valve**	**No**	**Postdilatation**	**Successful ring**	**Failed ring**
	**postdilatation**		**fracturing**	**fracturing**
**BioValsalva**, ***n*** **=** **3**				
#25	1	–	–	–
#27	2	–	–	–
**Perimount magna ease**, ***n*** **=** **2**				
#21	–	–	2	–
**MitroFlow**, ***n*** **=** **4**				
#23	2	–	–	–
#25	1	–	–	–
#27	–	1	–	–
**Mosaic**, ***n*** **=** **4**				
#23	1	–	1	–
#27	–	1	1	–
**Perimount**, ***n*** **=** **29**				
#21	–	1	1	3
#23	3	–	3	–
#25	5	2	–	3
#27	5	–	–	1
#29	2	–	–	–
**Trifecta**, ***n*** **=** **25**				
#19	–	2	–	–
#21	1	7	–	–
#23	5	5	–	–
#25	–	2	–	–
#27	1			
#29	2	–	–	–

In four procedures, a left main stem chimney stenting was performed for low left coronary artery ostia. In one case with small aortic root and low coronary distance, coronary stent-graft positioning over the prepositioned coronary wire failed, leading to fatal intraprocedural coronary occlusion.

There was one additional intra-hospital death for pneumonia in a patient with severe pulmonary disease.

Two minor (one access site hematoma and one pseudoaneurysm) and two major vascular complications (one bleeding for vascular closure device failure and stent-graft implantation and one access site bleeding/hematoma requiring transfusion) were observed.

Both major vascular complications qualified for a major bleeding complication according to VARC criteria. No life-threatening bleeding event occurred.

One perioperative nondisabling stroke was noted. One patient presenting in NYHA class IV for severe valvular regurgitation became hypotensive during induction of conscious sedation requiring CPR. After subsequent ViV-TAVR under chest compression, the patient experienced a disabling stroke.

### BVF

Successful BVF was achieved in eight patients, while seven valves could not be fractured (Table 2).

The time from SAVR to ViV-TAVR was 12.1 ± 3.5 years in successful BVF and 10.1 ± 3.1 years in failed attempts (*p* = 0.52).

Perimount valves with successful valve fracturing had been implanted in 1999, 2005, 2006, and 2007. Perimount valves with failed BVF had been implanted in 2007, 2008, 2009, 2010, and 2013. All Perimount valves with BVF attempts were the P2800 model.

The true-ID-to-outer-balloon-diameter ratio was 1.15 ± 0.06 in procedures with successful BVF and 1.12 ± 0.08 in failed BVF attempts (*p* = 0.443). The true-ID-to-outer-balloon-diameter ratio in cases not aiming for BVF was 1.06 ± 0.05 (*p* = 0.002). The mean duration of balloon expansion was 13.5 ± 3.5 s in procedures with successful and 17.3 ± 3.9 s in procedures with failed BVF (*p* = 0.128). None of the cases with unsuccessful BVF was caused by a balloon rupture.

No intraprocedural complication secondary to BVF, such as annular rupture or pericardial effusion, was observed.

### Hemodynamic Results

The baseline mean transprosthetic aortic valve gradient was 34.3 ± 12.5 mmHg, and the baseline mean effective orifice area (EOA) was 0.86 ± 0.3 cm^2^. Eighteen patients showed aortic regurgitation as the leading failure mode of the surgical aortic valves.

The mean transprosthetic gradient following ViV-TAVR was reduced to 12.6 ± 7.4 mmHg (*p* < 0.001) and EOA increased to 1.48 + 0.5 cm^2^ (*p* < 0.001). Mild paravalvular regurgitation was seen in nine patients (13%). No patient showed more than mild PVL. At discharge.

VARC-2-defined normal prosthetic valve function was found in 88.1% of the cases. VARC-2-defined device success was found in 86.6% of the cases.

**Figure 2:**
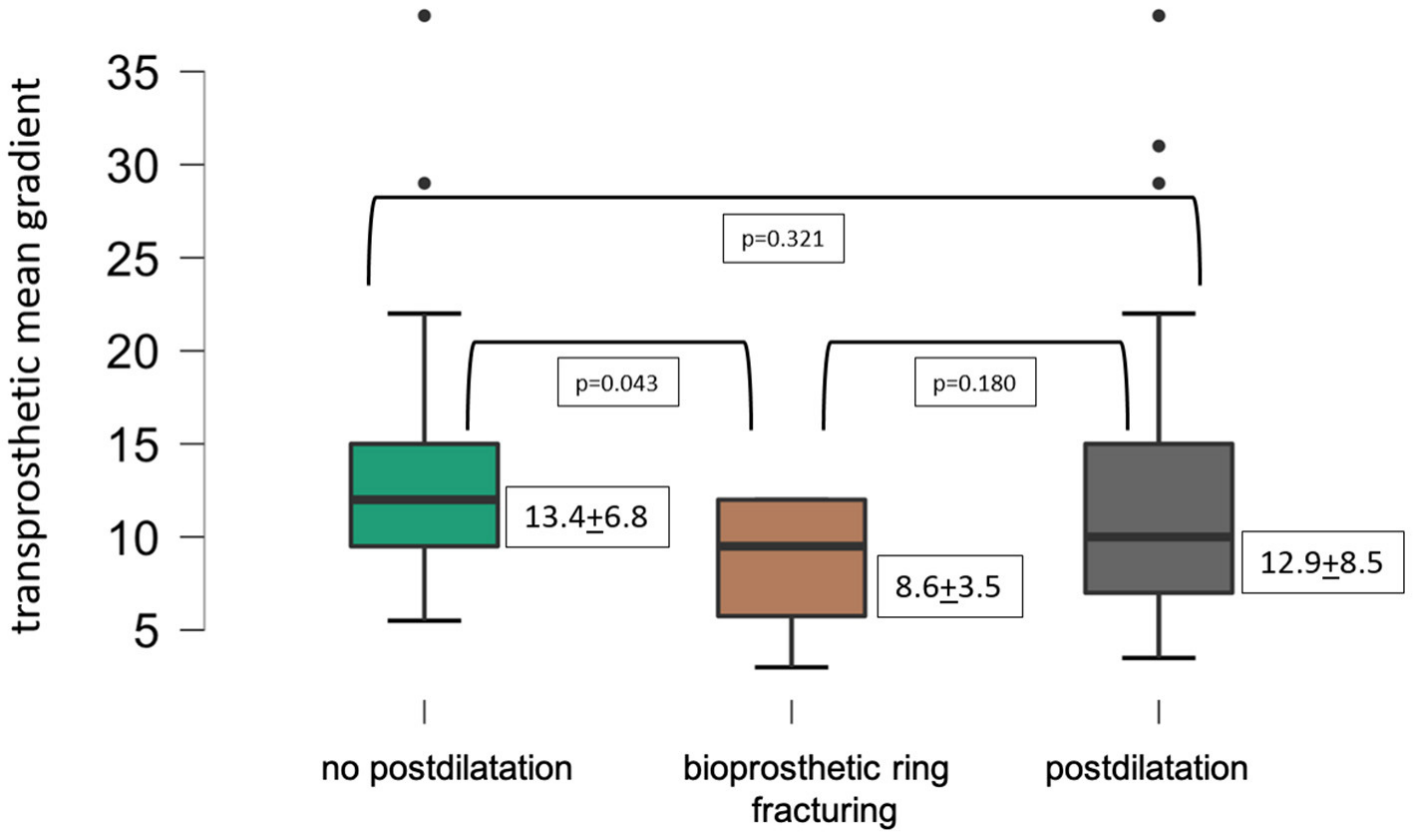
Mean transprosthetic gradient after ViV-TAVR.

Figure 2 displays lower transprosthetic gradients after successful BVF compared to cases with high-pressure postdilatation or without postdilatation.

### Small Surgical Valves

**Figure 3:**
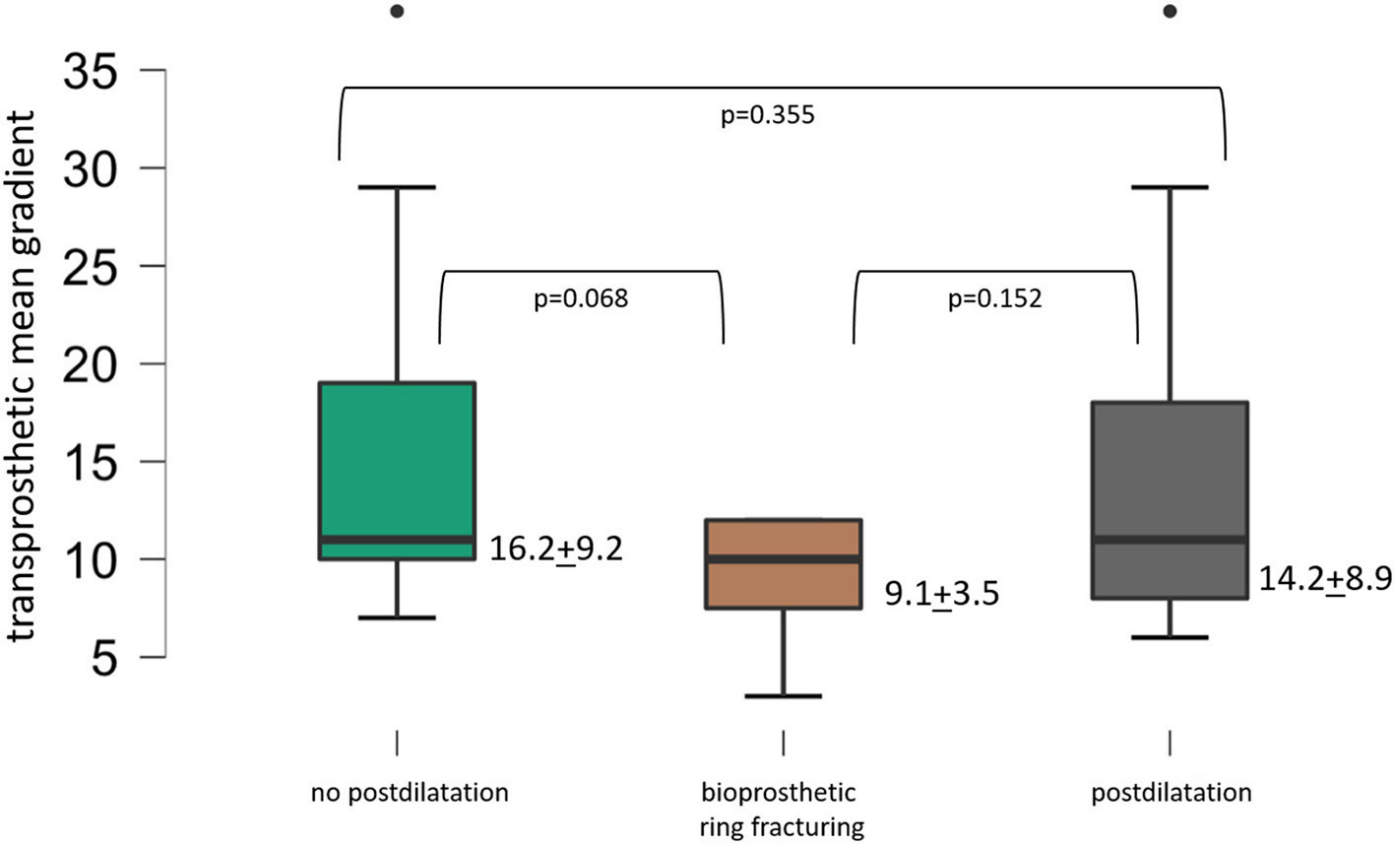
Mean transprosthetic gradient after ViV-TAVR with small surgical valves.

A small true ID ≤ 21 mm was found in 38 surgical valves. In 10 cases, BVF was attempted, with seven being successful. In 15 further cases, standard postdilatation was performed and 13 procedures were performed without postdilatation. Figure 3 displays lower transprosthetic gradients after BVF compared to procedures with standard postdilatation (*p* = 0.152) or without postdilatation (*p* = 0.068). A balloon-expandable THV was used in three cases; a self-expandable THV was used in 35 cases.

### Utilization of THVs

**Figure 4:**
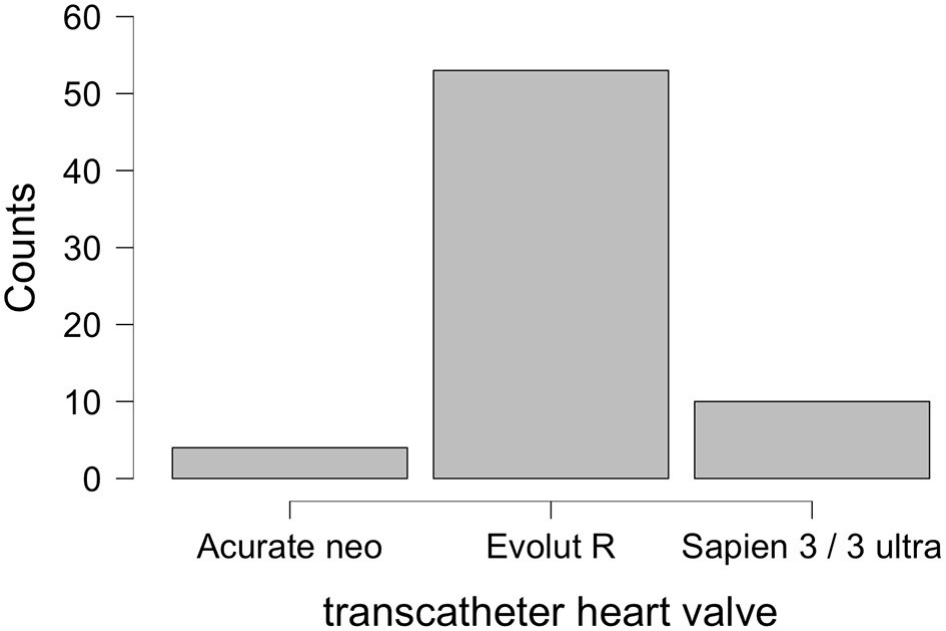
Distribution of transcatheter heart valves in 67 ViV-TAVR.

**Figure 5:**
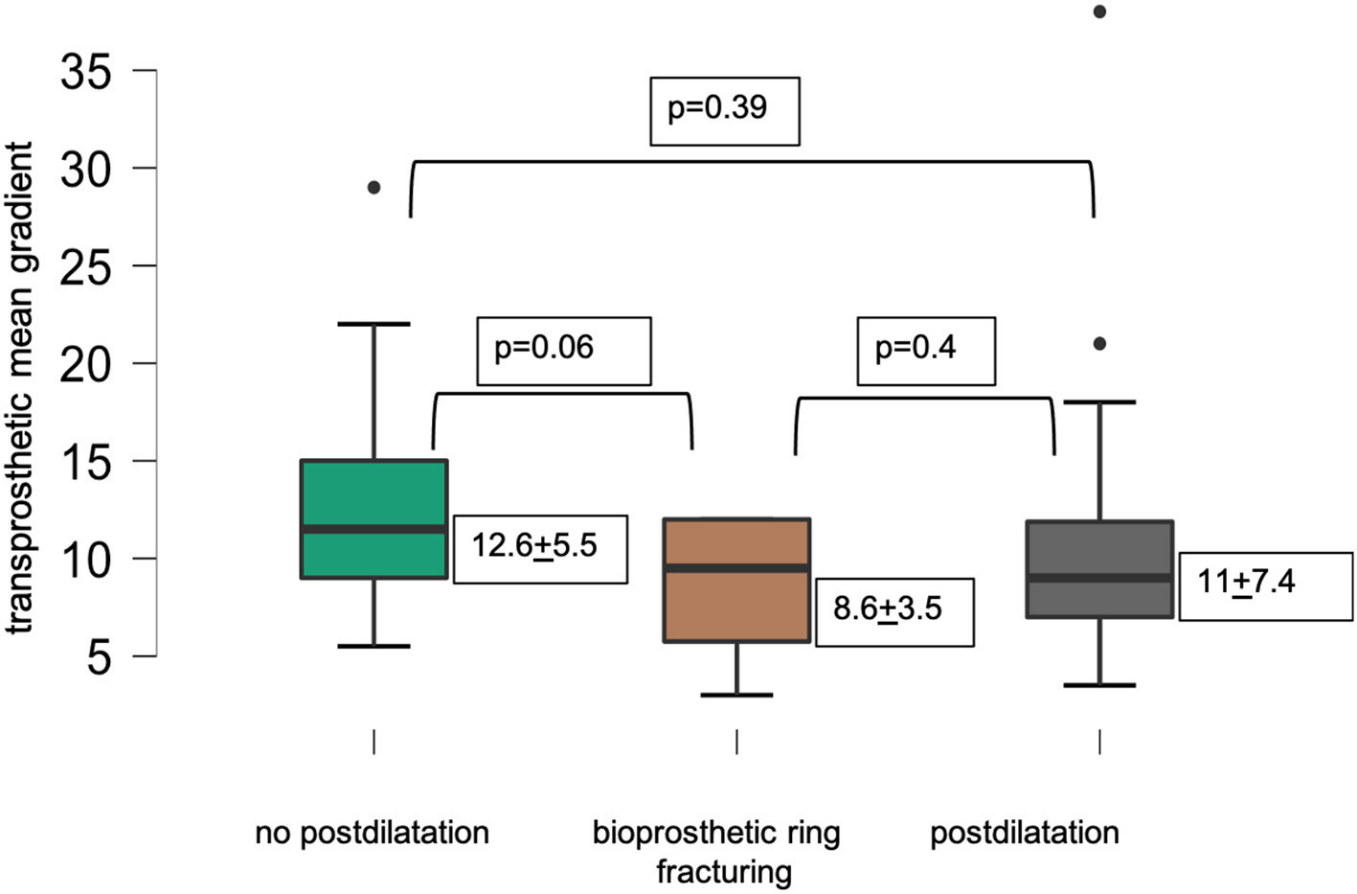
Mean transprosthetic gradient after ViV-TAVR excluding patients with a balloon-expandable THV.

Figure 4 displays the implanted THVs. The mean true ID of surgical valves was 21.2 ± 2.3 mm (range 17–27 mm) in ViV-TAVR with self-expandable, supra-annular THVs (*n* = 57) and 24.0 ± 2.5 mm (range 21–27 mm) in ViV-TAVR with balloon-expandable, intra-annular THVs (*n* = 10, *p* = 0.004). Mean transprosthetic gradient was 11.4 ± 6.2 after ViV-TAVR with self-expandable THVs and 20.4 ± 10.1 with balloon-expandable valves (*p* < 0.001). The most common THV for ViV-TAVR was the Evolut R with its supra-annular design. Rationales for use of balloon-expandable valves included transapical access and patient age with perspective of future valve-in-valve-in-valve procedures. Rationales for use of the Acurate THV included transapical access and ID of surgical valves ≤19 mm. The hemodynamic outcomes of patients receiving a self-expandable valve Evolut R or Acurate THV are summarized in Figure 5.

## Discussion

In an all-comers ViV-TAVR cohort of 67 patients, BVF was attempted in 15 procedures with eight being successful. In procedures with Perimount magna ease (Edwards Lifesciences, Irvine, USA) and Mosaic valves (Medtronic, Minneapolis, USA), we achieved a 100% success rate with BVF. In seven procedures with the Perimount standard surgical valve (Edwards Lifesciences, Irvine, USA), high-pressure-balloon dilatation failed to successfully fracture the bioprosthetic valve. BVF was predominantly used in ViV-TAVR with small surgical valves and achieved a lower mean transprosthetic gradient compared to procedures with standard postdilatation or procedures without postdilatation. In the whole cohort, statistical significance for transprosthetic gradients after ViV-TAVR was reached between BVF and procedures without postdilatation, but not between BVF and postdilatation. In the group of small surgical valves, bioprosthetic ring fracturing showed lowest transprosthetic gradients without reaching statistical significance. We found significantly lower transprosthetic gradients in procedures with self-expandable than in balloon-expandable THV.

Clinical experience with BVF is limited to case series (10, 13) and bench tests performed with dedicated surgical valves predominantly focusing on small valve sizes (5–7, 9). Technical failure of successful BVF includes rupture of the high-pressure balloon or “pinhole” defects (14).

*In vivo* studies showed that transprosthetic gradients after ViV-TAVR can consistently be lowered when applying BVF (6, 10). However, sometimes higher inflator pressures are required to achieve successful bioprosthetic ring fracturing (10) than had been reported from *in vitro* studies (5, 6, 8). This indicates that results from bench tests cannot be transferred directly to real-world ViV-TAVR procedures. However, determination of inflation-pressure thresholds for successful *in vivo* BVF among different surgical valves requires larger patient numbers and standardized balloon sizes for the individual surgical valve size.

In our study, the mean transprosthetic gradients were significantly lower in procedures with successful BVF compared to procedures without postdilatation. In procedures with postdilatation, the mean transprosthetic gradient was higher compared to BVF procedures, but statistical significance was not reached. Larger, randomized trials are needed to evaluate the potential benefits of BVF over standard postdilatation on transprosthetic gradients and outcome after ViV-TAVR.

Beside an improvement of transprosthetic gradients, BVF may be effective in treatment of paravalvular leakage along previously implanted surgical valves (15, 16).

Responsiveness of the standard Perimount valve (Edwards Lifesciences) to BVF was inconsistent in our hands. All 11 Perimount valves in which we attempted VF were of model 2800 and had equal fluoroscopic appearance. The dates of initial surgical valve implantation were similar distributed among those which were responsive/not responsive to BVF. The proposed distinction between “older” and “newer generation” Perimount valves (7) to foresee successful BVF did not apply in our patient cohort as we successfully attempted BVF in Perimount valves implanted before 2007. Especially, as the Perimount valve is a commonly implanted surgical bioprosthesis, the potential responsiveness to BVF needs further evaluation. Especially, the inflation-pressure threshold for successful BVF needs further evaluation. We used inconsistent oversizing (1–4 mm) of the balloons which might have an impact on the success of BVF attempts. The Perimount P 2800 valve has a similar fluoroscopic appearance as the Perimount Magna valve. As fracturing thresholds >20 atm have been reported for the Magna (6), a higher inflation pressure might have had achieved successful BVF in all P 2800 cases in our cohort.

There is ongoing discussion on the sequence of BVF and THV implantation. Performing BVF first will protect the THV from mechanical stress through the applied high pressure. One potential drawback of this procedural sequence may be a sudden and severe insufficiency of the surgical valve causing hemodynamic instability until implantation of the THV. A second potential drawback is embolization of valve material through balloon valvuloplasty before THV implantation. BVF after ViV-TAVR ensures optimal expansion of the THV and may lead to lower transvalvular gradients than BVF before THV implantation (13). Currently, it is unclear whether THV upsizing is dependent on the sequence with BVF first followed by ViV-TAVR or first ViV-TAVR followed by BVF.

Similar to the data published by Chhatriwalla et al. (10) and Allen et al. (13), we predominantly attempted BVF in small surgical valves. Optimizing transprosthetic gradients in this patient group has high clinical relevance as worse 1-year survival and long-term survival after ViV-TAVR corresponding to small surgical valves have been reported (3, 4).

Within the group of failed BVF, four out of seven surgical valves exhibited a true ID of 23 or 25 mm. So far, no data exist on the influence of surgical valve size on the success rate of BVF or likewise on the potential hemodynamic advantages of BVF in larger surgical valves. We applied BVF in larger valves especially in younger patients to optimize THV frame expansion.

No standardized protocol on the utilization of BVF has been established so far. Based on our clinical experience and the results from this study, we have standardized the procedure in order to achieve more conclusive data. We suggest using a high-pressure balloon at least 2 mm larger than the true ID of the surgical valve, an appropriate high-pressure inflator, and a centralized balloon position within the surgical valve. Prospective collected data are needed to assess the effect of this protocol on hemodynamics, survival, and long-term outcomes. A direct comparison of hemodynamic effects of ViV-TAVR using the same THV with or without VF or postdilatation within the same type of surgical valve is needed to further evaluate the roll of VF in ViV-TAVR.

Considerations prior to surgical aortic valve replacement should include potential strategies for later valve deterioration. Especially in small anatomy, surgical valves responsive to BVF should be preferred to facilitate optimal preconditions for future valve-in-valve treatment.

## Limitations

The study is limited by the retrospective and non-randomized design with small patient numbers. Invasive transvalvular gradients were recorded inconsistently and did not allow analysis of gradients before and after ViV-TAVR and BVF, respectively. The data have not been revised by an independent adjudication committee.

## Conclusions

BVF results in lower mean transprosthetic gradients after ViV-TAVR compared to procedures with standard postdilatation or without postdilatation. Inflation-pressure threshold for successful BVF in Perimount surgical valves, long-term hemodynamic outcome, and potential survival benefits need further evaluation.

## Data Availability Statement

The raw data supporting the conclusions of this article will be made available by the authors, without undue reservation.

## Ethics Statement

The studies involving human participants were reviewed and approved by Ethics commission, Faculty of Medicine, Technical University Munich. Written informed consent for participation was not required for this study in accordance with the national legislation and the institutional requirements.

## Author Contributions

HR is responsible for the design of the study, data collection and data analysis, and preparation of the manuscript. ME contributed to the data interpretation and revised the manuscript. RL contributed to data interpretation and revised the manuscript. All authors contributed to the article and approved the submitted version.

## Conflict of Interest

The authors declare that the research was conducted in the absence of any commercial or financial relationships that could be construed as a potential conflict of interest.
